# Running reorganizes the circuitry of one-week-old adult-born hippocampal neurons

**DOI:** 10.1038/s41598-017-11268-z

**Published:** 2017-09-07

**Authors:** Nirnath Sah, Benjamin D. Peterson, Susan T. Lubejko, Carmen Vivar, Henriette van Praag

**Affiliations:** 1 Neuroplasticity and Behavior Unit, Laboratory of Neurosciences, Intramural Research Program, National Institute on Aging, National Institutes of Health, Baltimore, MD 21224 USA; 2 Laboratory of Neurogenesis and Neuroplasticity, Department of Physiology, Biophysics, and Neuroscience, Center for Research and Advanced Studies of the National Polytechnic Institute, Mexico City, 07360 Mexico

## Abstract

Adult hippocampal neurogenesis is an important form of structural and functional plasticity in the mature mammalian brain. The existing consensus is that GABA regulates the initial integration of adult-born neurons, similar to neuronal development during embryogenesis. Surprisingly, virus-based anatomical tracing revealed that very young, one-week-old, new granule cells in male C57Bl/6 mice receive input not only from GABAergic interneurons, but also from multiple glutamatergic cell types, including mature dentate granule cells, area CA1-3 pyramidal cells and mossy cells. Consistently, patch-clamp recordings from retrovirally labeled new granule cells at 7–8 days post retroviral injection (dpi) show that these cells respond to NMDA application with tonic currents, and that both electrical and optogenetic stimulation can evoke NMDA-mediated synaptic responses. Furthermore, new dentate granule cell number, morphology and excitatory synaptic inputs at 7 dpi are modified by voluntary wheel running. Overall, glutamatergic and GABAergic innervation of newly born neurons in the adult hippocampus develops concurrently, and excitatory input is reorganized by exercise.

## Introduction

Adult hippocampal neurogenesis is considered to play a role in memory function and mood^1–3^. The development and integration of adult-born neurons follows a sequence of morphological and physiological events that extends over several weeks^4, 5^. Initially, the cells lack processes and are synaptically silent. The earliest input to new granule cells (GCs) is considered to be from γ-aminobutyric acid (GABA)ergic interneurons^6–8^. GABAergic transmission is excitatory during the first two weeks^6, 8^ and then switches to inhibitory as the new GCs become morphologically more mature with dendritic and axonal processes^9^. Around two weeks, the cells reportedly begin to receive innervation from glutamatergic mossy cells^10, 11^, followed by input from the entorhinal cortex during the third and fourth week^5, 12^. Thus, the current consensus is that GABAergic connectivity precedes glutamatergic innervation of new neurons in the adult hippocampus.

N-Methyl-D-aspartic acid receptors (NMDAR) are known to regulate prenatal neuronal development and connectivity^13, 14^. However, their role in the maturation and survival of adult-born neurons remains unclear. *In vivo*, administration of NMDA reportedly reduced adult hippocampal neurogenesis, while treatment with a NMDA antagonist increased new neuron number^15^. However, knockout of NMDAR1 in individual newborn neurons impacted their survival^16, 17^. Furthermore, in tissue culture experiments it was shown that application of the excitatory neurotransmitter glutamate to adult neural progenitor cells increased neuronal differentiation and excitatory synaptic connectivity^18^, suggesting that NMDAR respond to early glutamatergic innervation^10, 11, 19^. In addition, application of a high concentration of NMDA in acute hippocampal slices resulted in dendritic beading, indicative of functional NMDAR, in some immature neurons at 7 dpi^17^, providing support for the possibility that glutamatergic innervation may contribute to the initial wiring of new granule cells.

Previous studies have used pro-opiomelanocortin (POMC)-GFP and doublecortin (DCX)-EGFP transgenic mice to determine early inputs to new neurons^11, 19, 20^, however, the expression of these markers extends over several weeks in adult-born GCs^21–23^. Considering that glutamatergic, GABAergic and cholinergic signaling have been implicated in the regulation of adult neurogenesis^8, 17, 24^, it is important to determine the nature of the initial afferent inputs to adult-born neurons. In the present study, modified rabies virus^25^ was combined with retroviral labelling^12, 26^ to uncover the circuitry of very young (7 dpi) adult-born GCs in control and runner mice. Our data show that at 7 dpi immature GCs receive innervation from inhibitory interneurons and cholinergic basal forebrain cells, as well as robust input from multiple intra-hippocampal glutamatergic cell types, including mossy cells, mature GCs and area CA1-3 pyramidal cells. Consistently, patch-clamp recordings show that at 7–8 dpi adult-born GCs are activated tonically by ambient NMDA and exhibit NMDAR-mediated synaptic responses evoked by electrical or optogenetic activation of the granule cell layer. Our results reveal that young new dentate granule cells receive excitatory glutamatergic innervation just as early as GABAergic afferents. Moreover, we show that glutamatergic, but not GABAergic, inputs to adult-born granule cells are modified by running. These findings add new dimensions to our understanding of the maturation and functional integration of newly born neurons in the adult brain.

## Results

### Identification and quantification of inputs to one-week-old granule cells

**Figure 1:**
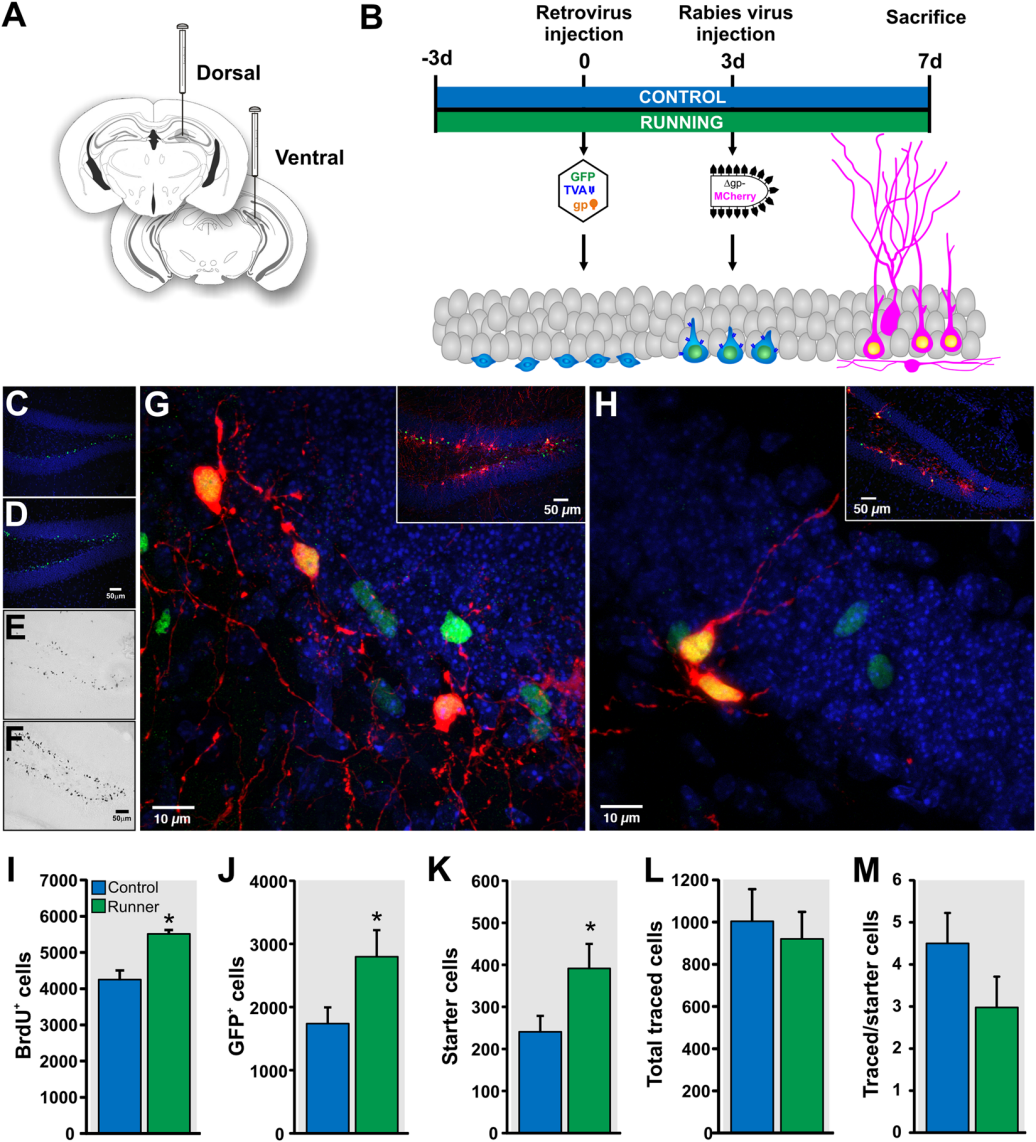
Circuitry tracing of one-week-old dentate granule cells in control and running mice. (**A**) Dorsal and ventral dentate gyrus injection of retrovirus (RV-SYN-GTRgp) and rabies virus (EnvA-ΔG-MCh). (**B**) Timeline of the experiment. (**C**,**D**) Photomicrographs of coronal sections derived from (**C**) control and (**D**) runner mice showing retrovirally infected cells expressing nuclear green fluorescent protein (GFP^+^) in the dentate gyrus. (**E**,**F**) Photomicrographs showing BrdU^+^ cells in coronal sections derived from (**E**) control and (**F**) runner mice. (**G**) Representative photomicrograph showing “starter cells” [retrovirus expressing nuclear GFP^+^- rabies virus expressing cytoplasmic MCh^+^ double labelled cells (green + red = yellow)] and retrovirally infected cells only (nuclear GFP^+^ only). Inset shows overview of the labeling. Nuclei were stained with 4′,6-diamidino-2-phenylindole (DAPI), blue. (**H**) Photomicrograph of a coronal section derived from a mouse injected with retrovirus missing Rgp (RV-SYN-HT) followed by rabies virus (EnvA-ΔG-MCh) in the dentate gyrus at the same inter-injection interval. Rabies virus infection did not spread beyond the “starter cells” (GFP^+^ - MCh^+^). Inset shows overview of the labeling. (**I**–**K**) Running increased (**I**) BrdU^+^ cell number, (**J**) GFP^+^ cell number and (**K**) “Starter cells” (GFP^+^ - MCh^+^) number in the dentate gyrus as compared to controls. (**L**) Running did not modify the total traced cell (MCh^+^ only) number. (**M**) Ratio of connectivity between traced and starter cells was not significantly different between controls and runners. *P < 0.05. Figure 1A: the drawings of the brain sections were modified from ref. 71, with permission from Elsevier to the Nature Publishing Group to publish these images under an Open Access license.

To study the initial wiring of adult-born GCs and to assess whether their afferent connectivity is modified by running, we applied the TVA-EnvA trans-synaptic tracing method^25^, a powerful strategy to assay neural circuits^12, 26–28^. Specifically, a retroviral vector (RV-SYN-GTRgp) expressing nuclear green fluorescent protein (GFP), TVA receptor and rabies virus glycoprotein (Rgp) driven by the neuron-specific synapsin promoter was used to selectively infect dividing cells, followed by the injection of EnvA-pseudotyped rabies virus lacking Rgp and expressing MCherry (EnvA-ΔG-MCh). Mice housed with (*n* = 8) or without a running wheel (*n* = 8) were injected with RV-SYN-GTRgp into the dorsal and ventral dentate gyrus to label proliferating neural progenitor cells (Fig. 1A,B). Three days later, mice received EnvA-ΔG-MCh rabies virus into the same dentate gyrus areas. This pseudotyped rabies virus selectively infects new neurons expressing the TVA receptor (Fig. 1B). Mice were perfused four days thereafter and sections were analyzed for expression of fluorescent reporters: GFP and MCh in the new neurons (‘starter cells’ that are the origin of the trans-synaptic tracing) and MCh only in traced cells (direct inputs to the adult-born neurons).

First, we determined whether running increased granule cell proliferation at 7 dpi. Histological analysis showed that the number of GFP^+^ GCs was significantly increased in runner mice as compared to controls (CON, 1738 ± 255 *vs* RUN, 2798 ± 420, *t*_(14)_ = 2.15, *P* < 0.05; Fig. 1C,D and J). Similarly, running significantly increased the number of bromodeoxyuridine (BrdU) labeled cells in a separate cohort of animals (CON, 4250 ± 255; *n* = 6 *vs* RUN, 5513 ± 111; *n* = 4, *t*_(8)_ = 3.53, *P* < 0.007; Fig. 1E, F and I). Dorsal-ventral distribution analysis showed that running increased cell genesis in the dorsal dentate gyrus, consistent with previous research^29^ (Supplementary Figure 1).

Next, we quantified the number of GFP^+^ cells that were dually infected with EnvA-ΔG-MCh (GFP^+^ - MCh^+^), which are called “starter cells”, as they are the origin of the trans-synaptic tracing^5, 12, 25^ (Fig. 1G). Starter cell number was significantly higher in runner mice as compared to controls (CON, 241 ± 38; RUN, 392 ± 58; *t*_(14)_ = 2.17, *P* < 0.05, Fig. 1K). Traced cells were observed in cortical and subcortical areas. The total number of presynaptic traced cells (MCh^+^ only; CON, 1004 ± 151; RUN, 920 ± 128; *t*_(14)_ = 0.42, *P* > 0.05; Fig. 1L), as well as the overall ratio of connectivity between the traced cells and the starter cells did not differ between groups (CON, 4.5 ± 0.7; RUN, 2.9 ± 0.7; *t*_(14)_ = 1.6, *P* > 0.05; Fig. 1M). As a control for possible non-specific rabies virus labeling, retrovirus missing Rgp (RV-SYN-HT), which is necessary for rabies virus complementation and virus spread to presynaptic neurons^25^, was injected into the dentate gyrus and was followed by EnvA-ΔG-MCh rabies virus three days thereafter (*n* = 3). We observed immature new neurons expressing GFP and GFP - MCh, but no traced cells expressing MCh only (Fig. 1H), consistent with previous research with a longer inter-injection interval^12^.

**Figure 2:**
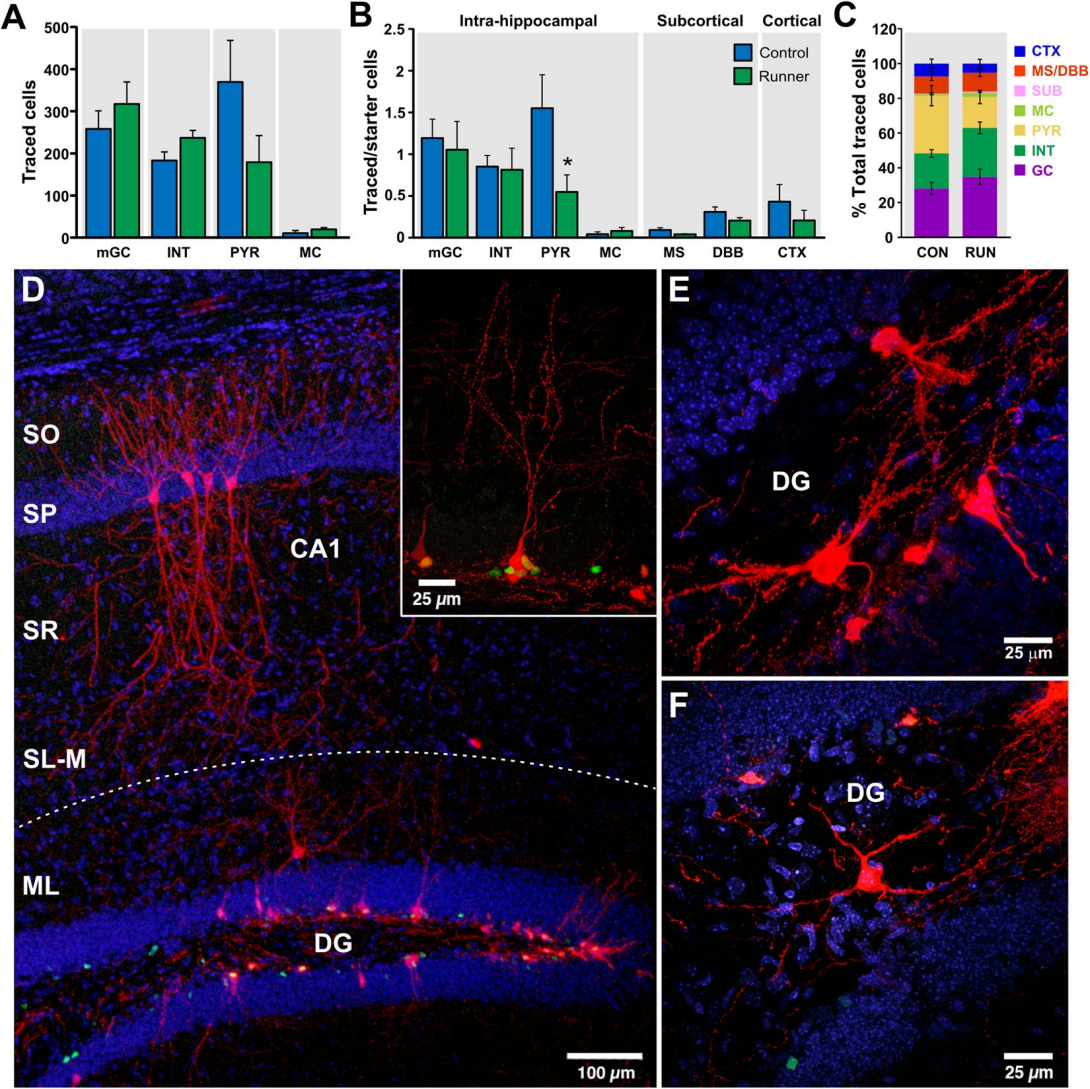
Monosynaptic inputs to one-week-old adult-born granule cells. (**A**) Running did not modify the numbers of intra-hippocampal cells [mature granule cells (mGC), interneurons (INT), pyramidal cells (PYR) and mossy cells (MC)]. (**B**) Ratio of connectivity of intra-hippocampal (mGCs, INT, PYR and MC), subcortical [medial septum (MS) and diagonal band of Broca (DBB)] and cortical [somatosensory, auditory and visual cortex (CTX)] cells to one-week old adult-born GCs. The ratio of connectivity from PYR to starter cells was significantly modified by running. (**C**) Percentage of contribution per cell type to the initial wiring of immature granule cells in controls and runners. (**D**) Representative photomicrograph showing GFP^+^ cells (green), “starter cells” (nuclear GFP^+^-cytoplasmic MCh^+^; yellow nucleus) and hippocampal traced cells (MCh^+^ only, red), including traced CA1 pyramidal cells (PYR) with their characteristic pyramidal shaped soma and apical and basal dendritic arborizations, and a traced interneuron localized in the molecular layer (ML) of the dentate gyrus (DG). Inset shows a traced mature granule cell (mGC) characterized by its elliptical cell body and its apical dendritic tree projecting throughout the entire ML of the dentate gyrus. (**E**) Traced mossy cells (MC) localized in the hilus of the dentate gyrus, with dendritic branches covered by large thorny excrescences. (**F**) Traced interneuron localized in the hilus of the dentate gyrus. Nuclei were stained with DAPI (blue). SL-M, *stratum lacunosum-moleculare*; SR, *stratum radiatum*; SP, *stratum pyramidale*; SO, *stratum oriens*, SUB, subiculum. *P < 0.05.

Analysis of the presynaptic traced cells per brain area showed that the majority of traced cells were intra-hippocampal cells (CON, 827 ± 121; RUN, 755 ± 92; Fig. 2). Based on their morphology and anatomical localization^12, 30, 31^, the traced hippocampal cells were identified as glutamatergic mature granule cells (mGCs), pyramidal cells (PYR), mossy cells (MC), and GABAergic interneurons (INT) (Fig. 2A,D–F). Glutamatergic mGCs were identified by their elliptical cell body localized within the granule cell layer and their apical dendritic tree projecting throughout the entire molecular layer (Fig. 2D, inset). The traced glutamatergic MC were localized in the hilus, identified as multipolar neurons with dendritic arborizations covered by large thorny excrescences (Fig. 2E). Glutamatergic PYR were localized within the pyramidal cell layer of the Cornu Ammonis (CA) areas CA1, CA2 and CA3, ipsi- and contra-lateral to the injection site, and were identified by their classic pyramidally shaped soma and their apical and basal dendritic arborizations (Fig. 2D). Different types of traced GABAergic INT were localized in the dentate gyrus (hilus and molecular layer) and area CA3, and they were identified by the localization of the soma, their dendritic and axonal arborization, and the lack of dendritic spines (Fig. 2D and F).

Quantitative analysis showed that the majority of intra-hippocampal presynaptic inputs come from glutamatergic mGCs and PYR, as well as GABAergic INT (Fig. 2A,C). There were only a few traced mossy cells ipsilateral to the site of virus injection and none were detected contralaterally. Interestingly, PYR were observed both ipsi-and contralateral to the injection site. In addition, the ratio of connectivity from PYR to starter cells was significantly modified by running (CON, 1.55 ± 0.4 *vs* RUN, 0.55 ± 0.2; *t*_(14)_ = 2.23, *P* < 0.05; Fig. 2B). Running did not change the ratio of connectivity from mGCs (CON, 1.2 ± 0.2; RUN, 1.05 ± 0.3; *t*_(14)_ = 0.34; *P* > 0.05) and INT (CON, 0.85 ± 0.13; RUN, 0.81 ± 0.26; *t*_(14)_ = 0.12; *P* > 0.05) to starter cells (Fig. 2B).

**Figure 3:**
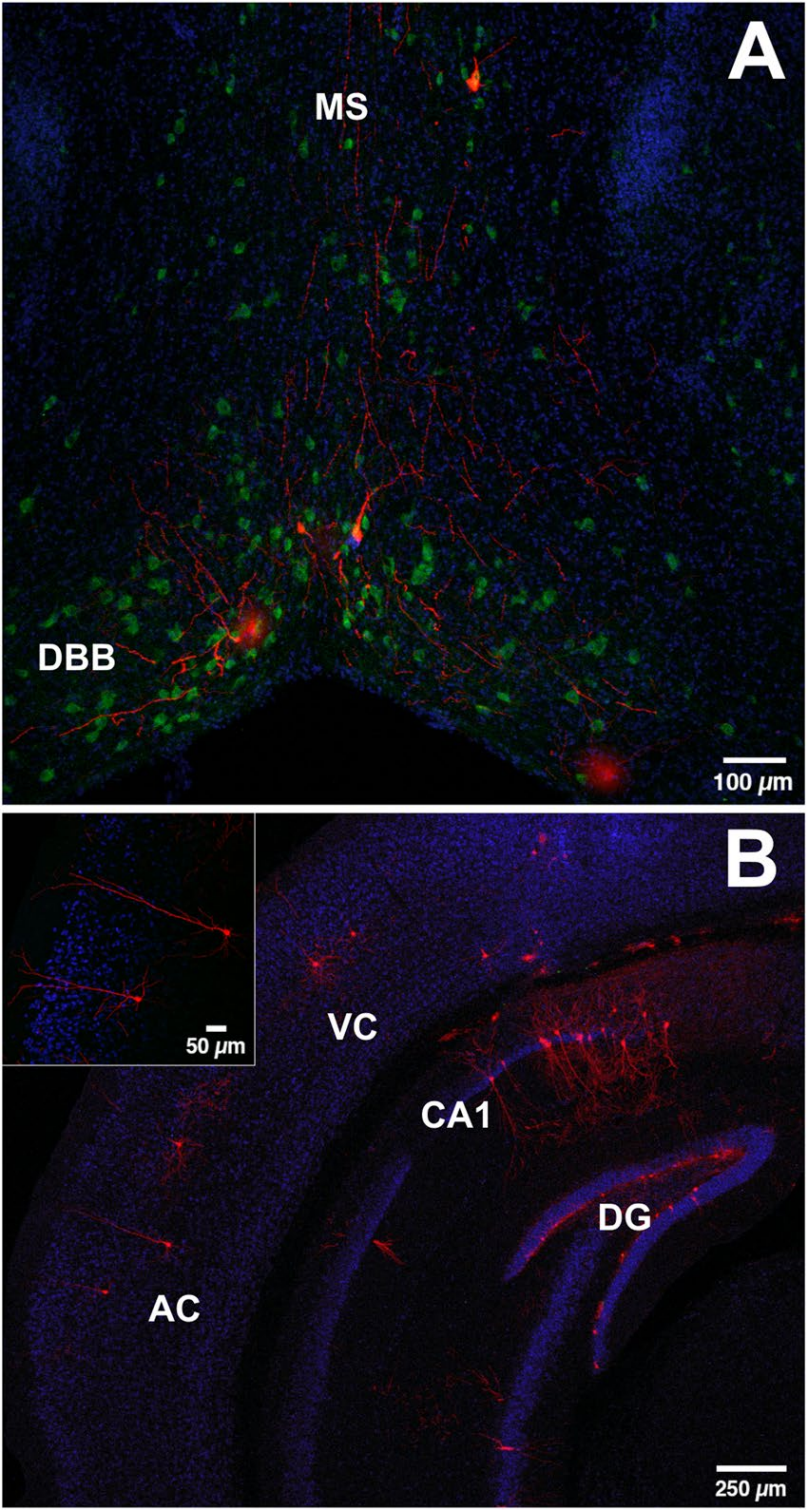
Subcortical and cortical connectivity to immature adult-born granule cells. (**A**) Coronal section derived from a control mouse showing traced cells expressing MCh (red) in the medial septum (MS) and horizontal nucleus of the diagonal band of Broca (DBB). MCh^+^ cells are immunoreactive for choline-acetyl-transferase (ChAT, green). Nuclei were stained with DAPI, blue. (**B**) Photomicrograph of a coronal section stained for the neuronal marker NeuN (blue), showing traced cells expressing MCh (red) in the visual (VC) and auditory (AC) cortex. Inset shows traced auditory cortex cells.

In addition to hippocampal cells, small populations of basal forebrain [medial septum (MS) and diagonal band of Broca (DBB)] and cortical cells (somatosensory cortex, auditory cortex and visual cortex), as well as sparse input from subicular cells, were observed (Figs 2C and 3). Trans-synaptic tracing in an additional cohort of older mice (8 weeks old) indicates that input from basal forebrain is not dependent of the age of the mice (Supplementary Figure 3). Running did not change the ratio of connectivity of MS (CON, 0.1 ± 0.02, RUN 0.04 ± 0.007, *t*_(14)_ = 1.82, *P* > 0.05; Fig. 2B), DBB (CON, 0.3 ± 0.06; RUN, 0.2 ± 0.04, *t*_(14)_ = 1.48, *P* > 0.05; Fig. 2B) and cortical cells (CON, 0.43 ± 0.2, RUN, 0.2 ± 0.12, *t*_(14)_ = 0.94, *P* > 0.05; Fig. 2B). Together, these results demonstrate that in addition to the GABAergic inputs, glutamatergic and cholinergic afferents are also involved in the initial wiring of new neurons.

### Running modifies the fine morphology of immature adult-born granule cells

**Figure 4:**
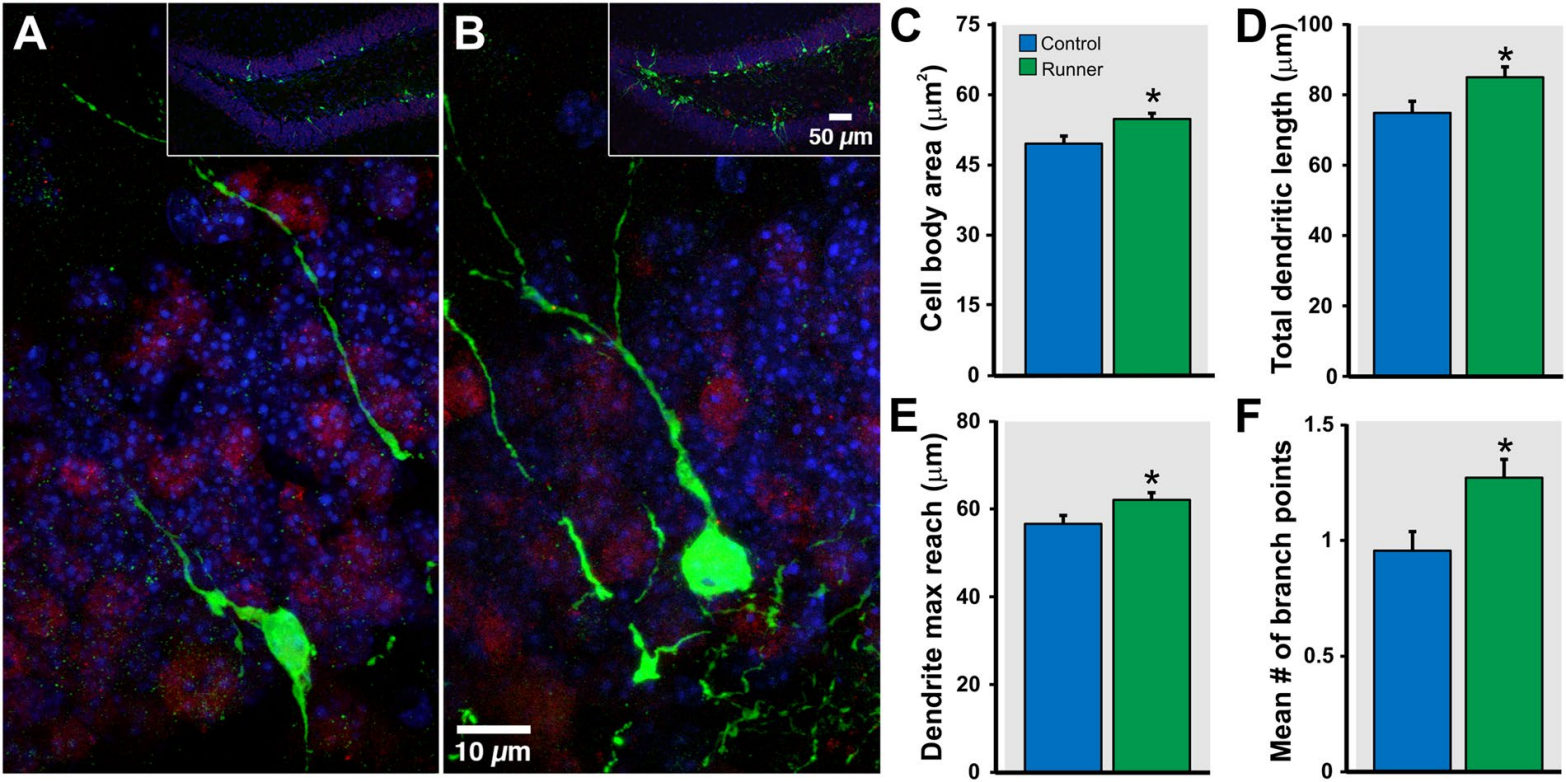
Running increases arborization of immature adult-born granule cells. (**A**,**B**) Retrovirally labeled GFP^+^ granule cells at 7 dpi from (**A**) control and (**B**) runner mice. Insets show the overview of the dentate gyrus. Morphometric analysis revealed that running significantly increases (**C**) cell body area, (**D**) total dendritic length, (**E**) dendritic maximum reach, and (**F**) mean number of branch points of new granule cells. Sections were co-labeled with neuronal marker NeuN (red). Nuclei were stained with DAPI, blue. *P < 0.05.

To evaluate whether the changes in neural circuitry elicited by short-term running are accompanied by alterations in the fine morphology of immature neurons, sedentary control and running mice were injected into the dentate gyrus with retrovirus expressing GFP^9, 32^. Specifically, the cell body and apical dendritic arbor of individual 7 dpi GFP^+^ neurons were traced and measured in controls (*n* = 137 cells from 4 mice) and runners (*n* = 183 cells from 4 mice). Only GFP^+^ neurons localized in the supra-pyramidal blade of the dentate gyrus and exhibiting an apical dendrite projecting to the molecular layer in dorsal hippocampal sections were traced. Morphometric analysis showed that running induces significant morphological changes in immature adult-born GCs. Specifically, the two-dimensional area of the cell bodies was significantly larger in running animals as compared to controls (CON, 49.6 ± 1.6 µm^2^ *vs* RUN, 54.6 ± 1.2 µm^2^; *t*_(318)_ = 2.53, *P* < 0.012; Fig. 4A–C). The total apical dendritic length, which is the sum of the length of each branch in the apical dendrite, was also significantly larger in running than in control mice (CON, 74.9 ± 3.3 µm *vs* RUN, 85.0 ± 2.9 µm; *t*_(318)_ = 2.29, *P* < 0.023; Fig. 4D). Additionally, maximum reach of the apical dendrite, defined as the length of the longest single branch of the apical dendrite from cell body to end, was significantly greater in voluntary running animals (CON, 56.6 ± 1.9 µm *vs* RUN, 61.7 ± 1.6 µm; *t*_(318)_ = 2.05, *P* < 0.041; Fig. 4E). Finally, the number of branch points in the apical dendrite can be used to quantify the complexity of its structure. Neurons from running animals had more nodes in their dendritic arbor on average than neurons from sedentary animals (CON, 0.96 ± 0.08 nodes *v*s RUN, 1.26 ± 0.08 nodes; *t*_(318)_ = 2.63, *P* < 0.009; Fig. 4F). The morphological modifications induced by running were accompanied by changes in intrinsic properties in new GCs. Electrophysiological recordings of GFP^+^ cells (7–8 dpi) showed significant differences in membrane capacitance (C_m_) and membrane time constant (Tau) but no changes in input resistance (R_in_) and resting membrane potential (RMP) with running (Table 1). In addition, no changes were observed in the amplitude of inward Na^+^ and outward K^+^ currents (Supplementary Figure 2).

**Table 1:** Intrinsic properties of young GCs.

	Control (*n* = 22)	Runner (*n* = 22)	*P-value*
RMP (mV)	−35.16 ± 2.43	−37.66 ± 1.43	0.38
R_in_ (GΩ)	3.61 ± 0.27	3.57 ± 0.13	0.90
Tau (ms)	75.13 ± 5.66	99.64 ± 6.53	0.007*
C_m_ (pF)	21.18 ± 1.07	27.99 ± 1.60	0.001*

Electrophysiological properties of immature adult-born granule cells at 7 ± 1 dpi from control sedentary and running mice. Values are expressed as mean ± SEM; RMP, resting membrane potential; R_in,_ input resistance; Tau, membrane time constant; C_m_, membrane capacitance.

### Glutamatergic connectivity is functional and NMDA receptor dependent

The observation that very young granule cells receive innervation from glutamatergic cells (mGC, PYR and MC) and that this network is modified by running led us to examine whether the glutamatergic connectivity is functional and whether running may affect early excitatory transmission onto immature GCs. To assess the functionality of the glutamatergic connectivity, retrovirus was injected into the dentate gyrus of sedentary control and running mice, and one week thereafter whole-cell recordings were performed on GFP^+^ cells in acute hippocampal slices.

**Figure 5:**
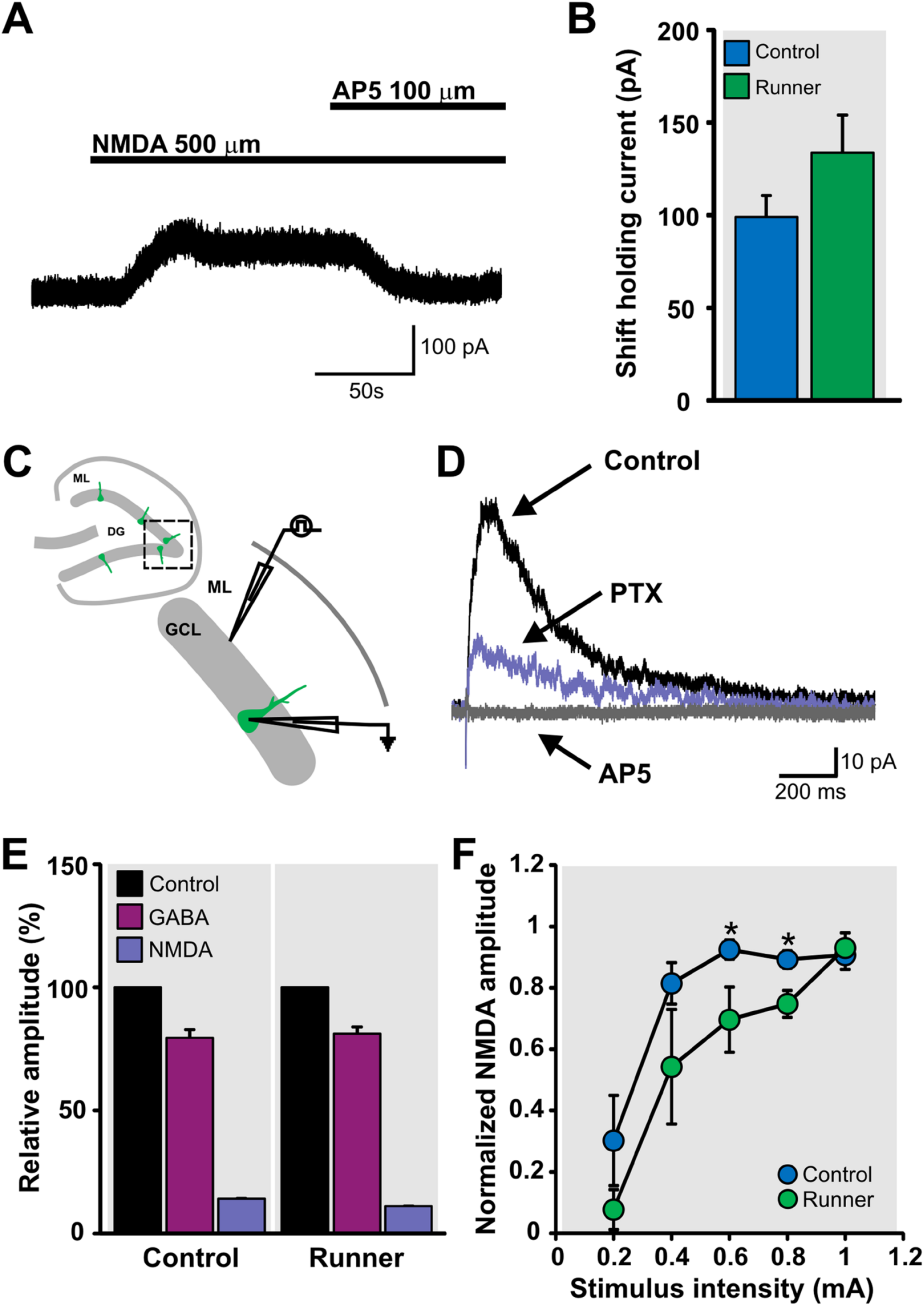
Glutamatergic connectivity is functional and NMDA receptor-dependent in adult born granule cells. (**A**) NMDA (500 μM) evokes an outward current (holding potential, V_h_ = +50 mV) that could be blocked by NMDA receptor antagonist AP5 (100 μM) in immature GCs (7dpi). (**B**) Running did not modify the shift in holding current induced by the application of NMDA (500 µM) in GFP^+^ cells at 7 dpi. (**C**) Schematic representation of the location of GFP^+^ recorded cells (dashed box) in acute hippocampal slices. Stimulation was performed in the inner molecular layer (ML) and whole-cell recordings were obtained from GFP^+^ at V_h_ = +50 mV. (**D**) Representative traces of evoked postsynaptic responses under control conditions (normal ACSF; black trace). Application of picrotoxin (PTX), an antagonist of GABA_A_ receptors, reduced the amplitude of the postsynaptic current (purple trace). The remaining current was completely blocked by AP5 (gray trace), an antagonist of the NMDA receptor. (**E**) Running did not modify the percentage of GABA_A_R-mediated and NMDAR-mediated currents. (**F**). Input-output curve of evoked NMDAR-mediated responses of GFP^+^ cells from control and runner mice. Significant differences between groups are observed at 0.6 and 0.8 mA stimulus intensity. *P < 0.02.

We first determined whether GFP^+^ cells express functional NMDA receptors (NMDAR). GFP^+^ cells recorded under whole-cell voltage-clamp (holding potential: V_h_ = +50 mV), in the presence of tetrodotoxin and specific antagonists of GABA_A_, GABA_B_ and AMPA receptors, were challenged with bath application of NMDA (500 μM). All of the recorded GFP^+^ cells responded to NMDA application with an outward current that was blocked by AP5 (100 μM), a specific antagonist of NMDAR (Fig. 5A,B). Running did not modify the amplitude of the outward NMDA current (CON, 99.0 ± 11.6 pA *vs* RUN, 133.9 ± 20.2 pA; *t*_(15)_ = 1.44, *P* > 0.17; Fig. 5B). These results demonstrate that immature GCs express functional NMDAR.

Next, we investigated whether there are functional glutamatergic synapses onto the immature GCs. We electrically stimulated the inner molecular layer of the dentate gyrus and recorded synaptic responses of GFP^+^ cells (V_h_ = +50 mV; Fig. 5C). Synaptic stimulation evoked postsynaptic currents with a peak amplitude of 74 ± 15.9 pA, which was blocked by picrotoxin (PTX; 20 μM), a specific antagonist of the GABA_A_ receptor (GABA_A_R), in immature adult-born GCs from acute slices derived from control mice. A remaining AP5-sensitive postsynaptic current (15.1 ± 3.3 pA peak amplitude) was observed in 18 of 27 cells, suggesting the presence of functional NMDA-mediated synapses in immature GCs (Fig. 5D). Moreover, electrical stimulation did not evoke any response at holding potentials of +50 mV and −70 mV after blocking GABA_A_ and NMDAR, suggesting the absence of functional AMPA receptors (10 neurons from 5 mice). From the total current (V_h_ = +50 mV), 79.4 ± 2.7% was GABA_A_R-mediated current and 13.8 ± 2.9% NMDAR-mediated current (Fig. 5E). Running did not modify the percentage of GABA_A_R-mediated (81.2 ± 3.6%, *t*_(15)_ = 0.39, *P* > 0.7) and NMDAR-mediated currents (10.8 ± 2.6%, *t*_(15)_ = 0.76, *P* > 0.46; Fig. 5E). In addition, the percentage of cells that exhibited evoked NMDA current was not changed by running [CON, 66.6% (18 of 27 cells) *vs* RUN, 81.8% (18 of 22 cells); *P* > 0.33]. We computed their relative conductance based on the theoretical reversal potential of −75 mV and 0 mV for GABA and NMDA receptor currents, respectively. The relative GABAR conductance [71.04 ± 4.7% (control); 76.1 ± 4.6% (runner)] was significantly higher than NMDAR conductance [28.18 ± 4.7% (control); 23.2 ± 4.5% (runner)] in both groups (t_(7)_ = 4.54, *P* < 0.03, control; t_(8)_ = 5.77, *P* < 0.0004, runner). Together, these data suggest that GABAergic and glutamatergic NMDAR-mediated synaptic inputs develop concurrently and are functional in immature GCs.

To further study the functional properties of the NMDAR-mediated synapses, we examined the changes in evoked NMDAR-mediated responses at increasing stimulus intensities in GFP^+^ cells from control (CON) and runner (RUN) mice. Data was normalized to the maximal amplitude of NMDAR-mediated response in each cell and was averaged by group. One-way analysis of variance (ANOVA) with repeated measures revealed a significant difference between the input-output curves of the groups (CON, *n* = 7; RUN, *n* = 4; *F*_(1, 9)_ = 8.65, *P* < 0.016; Fig. 5F). Post-hoc comparisons showed significant differences between groups at 0.6 and 0.8 mA stimulus intensities (*P* < 0.02). Immature new GCs from runner mice required a higher stimulation intensity to reach the maximal NMDAR-mediated synaptic response. At 0.6 mA GFP^+^ cells from control mice reached 92.5 ± 3.1% of maximal NMDAR-mediated amplitude, whereas GFP^+^ cells from runners reached only 69.3 ± 9.5%. A similar difference was observed at 0.8 mA (CON, 89.3 ± 2.9% *vs* RUN, 75.9 ± 4.4% of maximal NMDAR-mediated amplitude). Together, these data show that running induces modifications in the functional properties of the NMDAR-mediated synaptic responses in very young new neurons.

### Optogenetic stimulation of dentate gyrus reveals synaptic input onto immature adult-born GCs

**Figure 6:**
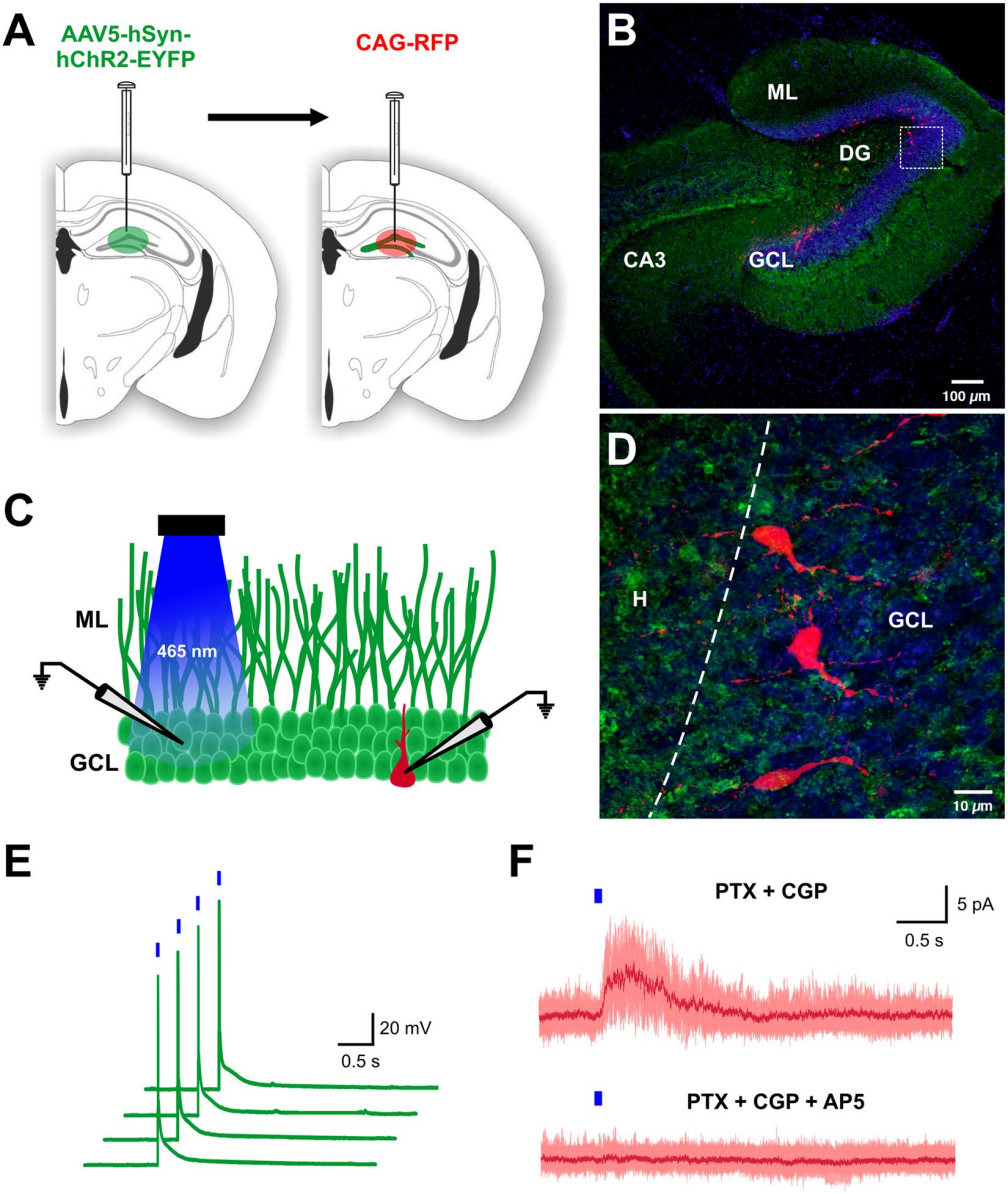
Optogenetic stimulation of dentate gyrus cells induces NMDAR-mediated responses in immature adult-born GCs. (**A**) Schematic representation of the viral injection. AAV5-hSyn-hChR2-EYFP viral vector was injected into the molecular layer of the dentate gyrus to express ChR2 in hippocampal neurons. Two to 3 weeks later, CAG-RFP retrovirus was injected into the same dentate gyrus to label dividing cells. (**B**) Photomicrograph of a horizontal section showing robust YFP expression in hippocampal neurons (green) and retroviral expression of RFP in immature adult-born granule cells (red) in the dentate gyrus. Nuclei stained with DAPI, blue. (**C**) Schematic representation of the experimental design. Granule cell layer (GCL) of the dentate gyrus was stimulated with brief blue light pulses (465 nm LED light, 10 ms, 0.1 Hz). Granule cell expressing YFP (green) and immature adult-born GCs expressing RFP (red) were electrophysiologically recorded. (**D**) High power photomicrograph of immature adult-born GCs (RFP^+^) in panel B (box) surrounded by YFP expressing fibers; dashed line between GCL and H, hilus. (**E**) Patch-clamp recordings under current-clamp configuration from a glutamatergic mature granule cell expressing ChR2-YFP. Brief light pulses trigger action potential. (**F**) Patch-clamp recordings under voltage-clamp configuration (V_h_ = +50 mV) of immature adult-born GCs (7 dpi). Optogenetic stimulation of the granule cell layer in presence of GABA receptor blockers (Picrotoxin [PTX] and CGP55845 [CGP]) evokes an outward current that is blocked by AP5, a selective antagonist of NMDA receptors. Figure 6A: the drawings of the brain sections were modified from ref. 71, with permission from Elsevier to the Nature Publishing Group to publish these images under an Open Access license.

To activate hippocampal neurons, we injected adeno-associated virus (AAV) expressing channel rhodopsin (ChR2) and yellow fluorescent protein [AAV5-hSyn-hChR2(H134)-EYFP] in the dentate gyrus. Two to three weeks later, retrovirus expressing red fluorescent protein (RFP) was injected into the same dentate gyrus to label dividing progenitor cells (Fig. 6A). Seven days later, patch-clamp recordings were performed from acute hippocampal slices. AAV injection resulted in robust YFP expression in granule cells, mossy cells and inhibitory neurons among other hippocampal neurons (Fig. 6B). Immature adult-born GCs (RFP^+^) were surrounded by YFP expressing fibers (Fig. 6D). To validate the functionality of the ChR2 expression, we performed patch-clamp recordings of glutamatergic mature granule cells expressing ChR2-YFP (Fig. 6C). Brief light pulses (465 nm LED light, 10 ms, 0.1 Hz) triggered action potentials (Fig. 6E). Next, to determine whether immature GCs (7 ± 1 dpi) receive glutamatergic inputs, we optically stimulated the granule cell layer of the dentate gyrus and recorded the synaptic response of immature GCs (RFP^+^) in the presence of GABA receptor blockers [Picrotoxin (20 µM), CGP55845 (1 µM)]. Optical stimulation elicited an outward current (peak 7.58 ± 2.44 pA; V_h_ = +50 mV) in 6 of 11 adult-born GCs, which was blocked by AP5 (100 µM), a selective antagonist of NMDA receptor (Fig. 6F). Thus, both optical and electrical stimulation evoked NMDAR-mediated synaptic responses in one-week-old adult-born GCs.

## Discussion

The conventional view is that inputs to adult-born granule cells are initially GABAergic, followed by glutamatergic innervation around the second week of development^6–8, 10, 11^. This is consistent with the concept that adult neurogenesis recapitulates neuronal development during embryogenesis^7, 33^. However, we show that one-week-old granule cells receive robust innervation from several glutamatergic cell types, including mature GCs, pyramidal cells and mossy cells, in addition to GABAergic afferents and cholinergic basal forebrain input. Together, these afferents orchestrate the early wiring of the adult-born neurons. Moreover, running modifies pyramidal cell innervation of these very young new neurons.

Excitatory synapses are typically formed on dendritic spines in mature neurons. NMDA receptor (NMDAR) activity is critical in regulating dendritic spine morphology and synaptic activity^34^. At 7 dpi, spines are absent in new neurons^9^ and previous studies indicate that electrical stimulation does not evoke glutamatergic currents at this developmental stage^7, 8^. However, we observed innervation from glutamatergic cells and evoked NMDAR-mediated responses induced by the application of NMDA, electrical and optogenetic stimulation. Our findings suggest that NMDAR can be activated in very immature new neurons. The potential explanation for the discrepancy between our results and previous studies^7, 8^ may be the experimental conditions utilized to evaluate the glutamatergic responses. We used an intracellular solution (cesium-based) that allowed us to unravel NMDAR-mediated responses (V_h_ = +50 mV). Indeed, research utilizing similar recording conditions shows that stimulation of the granule cell layer evokes NMDAR-mediated responses in immature GCs from pro-opiomelanocortin (POMC)-GFP mice^19^. Glutamatergic responses have been also observed in likely older immature GCs expressing doublecortin (DCX) in DCX-transgenic mice^20, 35^.

It has been shown that a high concentration of NMDA induces dendritic beading on the dendritic shaft of one-week-old GCs, consistent with the expression of functional NMDAR^17^. Therefore, it is possible that one-week-old GCs receive glutamatergic afferents directly onto their dendritic shafts. In embryonic cultures, transient excitatory synapses are formed on dendritic shafts, helping to initiate either the formation of dendritic protrusions or spines^36, 37^. Calcium influx through NMDARs may promote dendritic growth^17, 38, 39^ and the development of spines^37, 40–42^. It has been shown that a lack of NMDA receptors severely affects initial spinogenesis in adult-born neurons^16^. Since both GABA and glutamate are depolarizing at this developmental stage, synchronized activation of both inputs can initiate action potential firing^35^ and allow for calcium influx and downstream signaling^43^. Thus, early synaptic NMDAR-mediated inputs and GABAergic inputs onto immature GCs may promote initial dendritic growth and spine formation during the maturation process.

One-week-old adult-born GCs receive robust innervation from several glutamatergic cell types (including mature GCs, pyramidal cells and mossy cells). This may suggest that young new neurons receive stronger glutamatergic than GABAergic input. However, it is important to highlight that integration of glutamatergic and GABAergic synaptic inputs is not solely dependent on synapse number. It is powerfully influenced by cell morphology, including the passive membrane properties, the expression of voltage-gated ion channels (Na^+^ and K^+^), glutamatergic and GABAergic receptors as well as the location of the synapses (somatic or dendritic)^44^. Thus, GABAergic and glutamatergic inputs may have specific contributions to immature GC activity. Indeed, it has been shown that a few active interneurons (three or four) is sufficient to bring a small excitatory postsynaptic potential to firing threshold in immature GCs^35^. Further studies will be needed to understand the integration of these synaptic inputs.

Running increases dentate gyrus NMDAR gene expression and enhances synaptic plasticity mediated by NMDAR activation^45, 46^. While in our study the amplitude of evoked NMDAR-mediated synaptic responses in immature GCs did not differ between groups, we observed running-induced modifications in functional properties of the synaptic response, evaluated by changes in evoked NMDAR-mediated responses at increasing stimulus intensities. Specifically, immature GCs in acute slices derived from runner mice required a higher stimulation intensity to reach the maximal NMDAR-mediated synaptic response. This difference could be due to enhancement of synaptic contacts onto the more arborized dendrites of immature GCs from runner mice, while keeping the total postsynaptic NMDARs constant by redistribution over dendrites^47^. Indeed, it has been shown that shaft synapses have a higher density of postsynaptic glutamate receptors, and that this density decreases as the spines become longer^36, 48^. In agreement with this hypothesis, our results show that exercise enhanced morphological complexity which correlates with an increase in membrane capacitance (C_m_), without modifying the input resistance (R_in_). Thus, the activation of NMDA receptors by glutamatergic inputs at this very early time point (7 dpi) may mediate the formation of dendritic protrusions or spines. Indeed, we found overall increases in the cell body area and apical dendrite length, reach, and branching in GFP^+^ cells from running animals as compared to their sedentary counterparts. Previous work at later time-points after retroviral injection has also shown that running stimulates maturation of adult-born granule cells by increasing spine density, dendritic length and complexity^9, 49–52^. These running-induced morphological changes may expand dendritic surface area to potentially allow for a subsequent increase in synaptic connections.

Previously, we showed that one month of running modifies adult-born GC circuitry^26^. This coincides with the time-period of heightened excitability and synaptic plasticity of the newly born neurons^8, 53, 54^. It has been suggested that environmentally induced new neuron network changes are restricted to this time period^55^. However, our results show that running reorganizes new neuron circuitry much earlier than previously considered. Specifically, running reduced glutamatergic input from area CA1 pyramidal cells onto immature neurons. The input from area CA1 to new neurons may diminish during their development as innervation from this region to one-month-old neurons is sparse^26^. It is possible that the pyramidal cells that project to immature neurons are a transient additional source of glutamate or neurotrophins such as brain-derived neurotrophic factor (BDNF), and may be involved in the production of new neurons. Previous work indicates that there is an increase in hippocampal BDNF^56, 57^ and the tropomyosin receptor kinase B (TrkB) receptor^57^ after one week of exercise. These CA1 cells may be anatomically well-positioned to support running-induced enhancement of neurogenesis in the suprapyramidal blade of the DG^58^. Indeed, axons of area CA1 pyramidal cells project to the suprapyramidal layer of the DG and form functional connections with GCs in hippocampal slice cultures^59, 60^. Moreover, area CA1 inputs to newborn hippocampal neurons have been observed in a rat epilepsy model^61^. However, future studies will be needed to elucidate their specific role in the development of new neuron circuitry.

Mossy cells are considered to be the first glutamatergic synapses onto adult-born granule cells^10, 11^. However, we observed very few mossy cells innervating immature GCs at this developmental stage (with no innervation from the contralateral side), similar to previous research in mice^62^. Interestingly, in rats no mossy cell input was detected^61^. Our results suggest that glutamatergic input to new granule cells is not only provided by mossy cells, but also by several other types of glutamatergic cells, including mature GCs and pyramidal cells. It has been suggested that the tracing of mature GCs is due to pseudotransduction^62^. It is possible that a very short inter-injection interval renders non-proliferating cells susceptible to rabies virus infection and results in transgene expression^63^. However, injection of retrovirus missing rabies glycoprotein (which is necessary for rabies virus complementation and virus spread to the presynaptic neuron^25^) followed by EnvA-ΔG-MCh rabies virus at the same inter-injection interval shows only immature granule cells expressing GFP and GFP - MCh, but no traced cells expressing MCh only, supporting our findings and consistent with similar experiments conducted with longer inter-injection intervals^12, 61^. In agreement with our results, a recent study utilizing a viral-genetic tracing approach shows that dentate granule cells receive direct input from other granule cells and pyramidal cells^64^.

In addition to GABAergic and glutamatergic inputs, we observed a small input from basal forebrain cholinergic neurons. Our findings are consistent with a previous study describing the formation of axosomatic cholinergic synapses onto new GCs at 7 dpi^65^. Indeed, immature GCs express multiple types of acetylcholine receptors (AChR, α7, β2, m1 and m4) on their soma^66–68^ and the activation of AChR regulates the proliferation and survival of adult-born neurons^24, 67, 68^. Furthermore, exercise-induced increase of GC proliferation is abrogated by the ablation of the septal cholinergic system^68^. Thus, innervation by cholinergic neurons may play a key role in the integration of very young GCs into the already established hippocampal circuitry, even before dendritic maturation. In contrast with our results, a study shows that immature GCs (10 dpi) receive only local hippocampal inputs, mainly interneurons and very few mossy cells^62^. A potential explanation for this discrepancy may be the age of the mice (8–9 weeks old) used in the study. However, it is well known that cholinergic innervation to the hippocampus is already established around the second postnatal week^69^. In addition, we show in a cohort of 8-week-old mice, that basal forebrain input to new granule cells is not age-dependent. Another possible explanation is the efficiency of the trans-synaptic tracing system, as in this study a transgenic mouse model was used in which only a small proportion of immature granule cells express the TVA receptor^62^. Unfortunately, total numbers of starter and traced cells were not reported, precluding direct comparisons with our study.

Together, our data show that adult-born neurons are integrated into the hippocampal network by the first week of their development and receive both excitatory and inhibitory innervation. Overall, our research indicates that very young GCs are integrated into the DG circuitry through concurrent GABAergic, glutamatergic and cholinergic afferent innervation. Moreover, we provide converging evidence that the morphology, physiology and early network of new neurons can be reorganized by physical activity.

## Materials and Methods

### Animals

Male C57Bl/6 mice (Jackson Labs) 5–6 weeks old (*n* = 83) and 8 weeks old (*n* = 4) were individually housed. The 5–6 week old mice were randomly assigned to control or voluntary wheel running conditions. Running distance in silent spinner wheels (11.5 cm dia) was monitored as described previously^70^. Mice were housed with a 12 hour light-dark cycle (lights on at 6:00 a.m and off at 6:00 p.m.) with food and water *ad libitum*. Animals were maintained according to the National Institute of Health guidelines, and protocols for procedures were approved by the NIA Institutional Animal Care and Use Committee. All methods were performed in accordance with the relevant guidelines and regulations.

### Viral Vector Production

Retroviral vectors CAG-GFP, CAG-RFP, RV-SYN-GTRgp and RV-SYN-HT as well as rabies virus EnvA-ΔG-MCh were produced as previously described^12, 25, 32^. Specifically, retrovirus was produced by transient transfection (Lipofectamine, Invitrogen) of vector (7.5 μg), CMVGagPol (5 μg) and CMV-VSVG (2.5 μg) in 90% confluent 293T cells. Virus-containing supernatant was harvested 36 h later, filtered and concentrated by ultracentrifugation. Virus titers were estimated to be ~10 × E8 i.u. ml^−1^ by serial dilution into 293T cells. To produce EnvA-pseudotyped Δgp-mCherry rabies virus (EnvA-ΔG-MCh), a glycoprotein-gene-deleted rabies virus vector (Δgp-mCherry) was generated in which a mCherry (MCh) reporter gene was inserted into the locus encoding the rabies virus glycoprotein (kindly provided by E. Callaway, Salk Institute). The helper cell line, BHK-EnvARGCD, was infected with Δgp-mCherry, to produce EnvA pseudotyped rabies virus. Supernatants containing Δgp-mCherry rabies virus pseudotyped with EnvA were harvested 5 days later, filtered and concentrated by ultracentrifugation. Rabies virus titer was estimated to be ~1.2 × 10 E7 i.u. ml^−1^ and diluted for use to ~4 × 10E6 i.u. ml^−1^.

### Stereotaxic Surgery

After three days of housing in their respective conditions, mice were anaesthetized (Avertin 0.4 mg g^−1^ i.p.) and stereotaxic surgery was performed to deliver 1 μl of CAG-GFP or RV-SYN-GTRgp retrovirus into the right dorsal and ventral dentate gyrus using spatial coordinates relative to bregma as follows: Dorsal dentate gyrus, anterior-posterior (AP) = −2.10 mm; medial-lateral (ML) = 1.9 mm; dorso-ventral (DV) = −2.20 mm, and ventral dentate gyrus, AP = −3.10 mm; ML = 2.8 mm; DV = −3.20 mm. These coordinates were modified from the mouse brain atlas^71^ and adjusted for mice aged 5 weeks at the time of injection.

Mice injected with retroviral vector CAG-GFP were utilized for electrophysiology on 7–8 dpi. A subset of these mice was used for qualitative and quantitative histological analyses. Beginning on the day of stereotaxic surgery and lasting for three consecutive days, these mice also received a daily intraperitoneal injection of BrdU (50 mg kg^−1^) (Sigma-Aldrich) to quantify cell genesis in the two groups. BrdU was dissolved in 0.9% saline and filtered at 0.2 mm. These mice were anesthetized and perfused transcardially on 7 dpi as described below.

For trans-synaptic tracing mice injected with RV-SYN-GTRgp were anesthetized three days after retroviral administration to deliver rabies virus EnvA-ΔG-MCh (1 μl) into the same locations. Four days thereafter, on 7 dpi, animals were given an overdose of anesthetic and perfused transcardially with 0.9% saline at room temperature followed by cold 4% paraformaldehyde in 0.1 M PBS. After post-fixation for 24 h, brain tissue was equilibrated in 30% sucrose. Sequential coronal sections (40 μm) were taken using a freezing microtome through the rostral-caudal extent of the brain and stored in phosphate-buffer glycerol at −20 °C.

For optogenetic stimulation experiments, the viral vector AAV5-hSyn-hChR2(H134)-EYFP [University of Pennsylvania Vector Core with permission from K. Deisseroth (Stanford University, Stanford, CA)] expressing neuronal-specific channel-rhodopsin was injected into the suprapyramidal blade of the dentate gyrus in two locations (l µl volume per/location) using the following coordinates (relative to bregma): AP = −2.10 mm; ML = 1.9 mm; DV = −2.00 mm, and, AP = −3.10 mm; ML = 2.8 mm; DV = −3.00 mm. AAV-ChR2 injection was followed by retrovirus CAG-RFP injection 2–3 weeks later into the dorsal and ventral dentate gyrus, as described above. Mice were utilized for optogenetic experiments at 7–8 days after retrovirus injection.

### Immunohistochemistry, cell counts and morphological analysis

#### BrdU labeling and quantification

To label sections for BrdU-positive cells, we used the rat anti-BrdU antibody (1:200, AbD Serotec) with a biotinylated donkey anti-rat secondary antibody (1:250, Jackson ImmunoResearch Laboratories). Staining was completed using the ABC peroxidase complex (ABC Kit, Vector Laboratories) with the chromogen 3,3′-diaminobenzidine (Sigma-Aldrich) as described previously^70^. BrdU-positive cells in the dentate gyrus were counted in a one-in-six series of 40 µm sections (240 μm distance between the sections) using 20x objective light microscopy (BX51, Olympus) and the Stereo Investigator imaging software (MBF Bioscience). Counts were restricted to the hemisphere that was not injected with retrovirus. Total BrdU-positive cell numbers were obtained by multiplying the counts in the series by six.

#### Morphological analysis

Forty µm coronal sections, a one-in-six series derived from mice injected unilaterally with retrovirus CAG-GFP, were fluorescently stained for green fluorescent protein (GFP) and the neuronal marker NeuN. Antibodies for GFP (chicken polyclonal, 1:500; Aves Labs) and NeuN (mouse monoclonal, 1:100; EMD Millipore) were combined in TBS with 3% donkey serum and 0.1% Triton X-100 (Sigma-Aldrich). Secondary antibodies used were donkey anti-chicken AF488 (1:250, Jackson ImmunoResearch Laboratories) and donkey anti-mouse Cy3 (1:250, Jackson ImmunoResearch Laboratories). 4′,6-Diamidino-2-phenylindole, dihydrochloride (DAPI) (Molecular Probes) was applied to localize cell nuclei. Sections were imaged using confocal microscopy (FV1000 BX61WI, Olympus) with a 20x objective and step size of 1 µm. Imaging was focused on the suprapyramidal blade of the DG in dorsal hippocampal slices in subjects that had a minimum of 50 GFP^+^ cells in total. To compare the cell body, dendritic arborization, and dendritic reach of individual neurons from runners and sedentary animals, Z-series image TIFF files were merged using Slidebook software (Intelligent Imaging Solutions). Each individual neuron exhibiting either a single, non-branching apical dendrite or a single, branching apical dendrite, was traced using Neurolucida (MBF Bioscience). Each tracing was analyzed for cell body area, apical dendrite length, and branch points with Neurolucida Explorer software (MBF Bioscience). Total dendritic length was defined as the summed length of all branches in the apical dendrite. Apical dendrite maximum reach was defined as the length (in microns) of the longest single branch of the apical dendrite, measured from the cell body to end of the branch. Cells from running and sedentary animals were analyzed for each parameter. Results from individual cells were averaged by condition for comparison.

#### Retrograde tracing analysis

To quantify the number of newly born neurons and their afferent inputs, 1:6 series (240 µm apart) of coronal sections (40 µm) throughout the rostral-caudal extent of the brain were used. Sections were stained for GFP (1:1000, chicken polyclonal, Aves Labs) and red fluorescent protein (RFP, 1:1000, rabbit polyclonal, Rockland) and the corresponding fluorescent secondary antibodies (1:250, donkey anti-chicken Alexa Fluor 488; 1:250, donkey anti-rabbit CY3, Jackson ImmunoResearch). Nuclei were stained and visualized with DAPI.

#### Dentate Gyrus adult-born starter cells

To determine the number of starter cells (GFP^+^-MCh^+^) in the DG, confocal images (FV 1000MPE, Olympus), fifteen to eighteen z-planes at 1 µm intervals, were taken at 20x and quantified in a 1:6 series (240 µm apart) of coronal sections (40 µm) throughout the rostral-caudal extent of the brain. Total cell numbers were obtained by multiplying by six. Only mouse brains with starter cells throughout the entire dentate gyrus were taken for tracing analysis (8 of 19 of the control group and 8 of 17 of the running group).

#### Hippocampal traced cells

Within the hippocampus, traced cells (TC, MCh^+^ only), including mature granule cells (mGCs), interneurons (INT), pyramidal cells (PYR) and mossy cells (MC), were counted. In the DG, the mGCs and INT were separated based on location and morphology. Mature granule cells (mGCs) expressing MCherry (MCh) were identified morphologically by their elliptical cell body localized in the granule cell layer (GCL) and their characteristic cone-shaped tree of spiny apical dendrites. Interneurons (INT) were identified based on their location and morphology^30^, as described previously^26^. Mossy cells (MC) were identified by their location in the hilus of the DG and characteristic morphology, thorny excrescences covering the proximal ends of their long, thick dendritic branches^31^.

#### Distal traced cells

To evaluate the traced cells (TC) in other brain areas, sections were imaged at 4x using a fluorescent microscope (BX51, Olympus). Sections were reconstructed by stitching the images using CorelDraw. After reconstruction, sections were matched to the mouse brain atlas^71^ to determine the rostro-caudal distance from bregma. Next, images were taken with a 10x objective (BX51, Olympus) for detailed quantification of the traced cells. These cells were classified and counted based on the mouse brain atlas^71^ throughout the rostral-caudal extent of the brain by experimenters who were blinded to group identity of the samples. Total cell numbers were obtained by multiplying by six.

### Electrophysiology

One week after retrovirus injection, mice were anaesthetized and decapitated. Brains were removed into a chilled solution containing (in mM): 110 Choline-Cl, 2.5 KCl, 1.25 NaH_2_PO_4_, 25 NaHCO_3_, 25 glucose, 1 CaCl_2_, 7 MgCl_2_, 0.6 Na^+^ pyruvate, 1.3 Na^+^ ascorbate, and 3 kynurenic acid. Horizontal cortico-hippocampal slices (300 μm thick) were obtained from dorsal to mid-dorsal hippocampus and transferred to a chamber containing incubation solution (in mM): 125 NaCl, 2.5 KCl, 1.25 NaH_2_PO_4_, 25 NaHCO_3_, 2 CaCl_2_, 2 MgCl_2_, 20 glucose, 3 Na^+^ pyruvate and 1.3 Na^+^ ascorbate (pH of 7.4 and osmolarity of ~305–310 mOsm, when equilibrated with 95% O_2_ and 5% CO_2_). Slices were incubated at 34 °C for 10 min and stored at room temperature for >1 hour before patch clamp experiments. Recordings were carried out in artificial cerebrospinal fluid (ACSF) containing (in mM): 125 NaCl, 2.5 KCl, 1.25 NaH_2_PO_4_, 25 NaHCO_3_, 2 CaCl_2_, 1.3 MgCl_2_ and 20 glucose, equilibrated with 95% O_2_ and 5% CO_2_ (pH 7.4, osmolarity ~305 mOsm) at room temperature (~22 °C) using microelectrodes (6–8 MΩ) pulled from borosilicate glass. For intrinsic properties, pipettes were filled with K-based pipette solution containing (in mM): 130 K-gluconate, 7 KCl, 0.1 EGTA, 10 HEPES, 5 Mg-ATP, 0.5 Na-GTP and 10 Na-phosphocreatine (pH 7.4, adjusted with KOH; 290 mOsm). Cesium-based pipette solution containing (in mM): 125 CsOH, 125 gluconic acid, 7 CsCl, 10 HEPES, 0.1 EGTA, 5 Na-ATP, 0.5 Na-GTP, 10 Na-phosphocreatine (pH 7.4, adjusted with CsOH; 290 mOsm) was used to record electrically evoked synaptic currents. For the tonic NMDA current experiments, tetrodotoxin (0.5 μM; Tocris Bioscience), bicuculline methiodide (100 μM; Abcam), picrotoxin (100 μM; Tocris Bioscience), (2S)-3-[[(1S)-1-(3,4-Dichlorophenyl)ethyl]amino-2-hydroxypropyl](phenylmethyl)phosphinic acid hydrochloride (CGP 55845, 1 μM), 6-Cyano-7-nitroquinoxaline-2,3-dione (CNQX, 10 μM; Tocris Bioscience) and glycine (5 μM; Sigma-Aldrich) were bath applied. Electrical stimulation experiments were performed in ACSF and GABA_A_ receptor antagonists (bicuculline methiodide, 100 μM, picrotoxin, 100 μM), GABA_B_ receptor antagonist (CGP 55845, 1 μM; Abcam) and NMDA receptor antagonist (DL-2-Amino-5-phosphonopentanoic acid, 100 μM; Abcam) were used wherever specified.

Newborn neurons (7 ± 1 dpi) were identified by expression of GFP or RFP under epifluorescence and visualized with infra-red differential interference contrast video microscopy (Olympus BX51WI). Whole-cell patch-clamp recordings (Multiclamp 700B, Axon Instruments) were made from GFP^+^ or RFP^+^ cells localized in the crest of the dorsal to mid-dorsal hippocampal slices. Recordings were filtered at 2 kHz and digitized at 10–50 kHz (Digidata 1440 A and pClamp 10.4 Software; Molecular Devices). Series resistance was typically 10–30 MΩ. Ten - 20 sweeps of membrane potential deflections during a 1000 ms depolarizing pulse of 4 pA were averaged to calculate input resistance and membrane time constant. Input resistance was calculated as the ratio of steady-state voltage deflection and depolarizing current pulse (4 pA). Membrane time constant was estimated by fitting a single exponential to the initial 100 ms of membrane potential deflection and capacitance was calculated as the ratio of membrane time constant and input resistance. GABA/NMDA receptor currents from each cell were converted to conductance values using the equation:

$$g=I/(V-{V}_{rev}),$$

where *g* is conductance, *I* is the peak current amplitude, *V* is the holding potential (+50 mV) and *V*_*rev*_ is the reversal potential (GABA: −75 mV and NMDA: 0 mV)^72^.

All the experiments performed in voltage clamp mode were held at +50 mV. Tonic NMDA current was calculated as the difference between the baseline holding current (I_hold_) and I_hold_ in the presence of 500 μM NMDA. All point histogram plots were constructed and the peak current value was selected as I_hold_ in each experimental epoch. For the evoked synaptic responses, the stimulus (20μs, 0.2 Hz) was applied through a bipolar theta electrode filled with ACSF, positioned in the inner molecular layer ~200–250 µm away from the recording electrode (Fig. 5C). The evoked synaptic currents constitute an average of 3–5 repetitions per synaptic response. All signals were analyzed with Clampfit 10 Software.

### Optogenetics

Optical stimulation was achieved by transmitting light from a 465-nm Plexibright LD-1 LED module (Plexon, Dallas, TX, USA) via an optic fiber (core diameter: 200 µm) to the granule cell layer of the dentate gyrus, 250 µm away from the recording electrode. Square pulse stimulation of 10 ms was applied at 0.1 Hz using Digidata 1440A (Molecular Devices) and PlexBright LD-1 Single Channel LED Driver (Plexon, Dallas, TX, USA).

### Statistical analysis

Statistical analyses were carried out using Statview. Comparisons between control and running groups for histological and electrophysiological data were performed with Students t-test or One-way analysis of variance (ANOVA) with repeated measures followed by Fisher’s post-hoc test. Proportion comparisons between groups were performed with Fisher’s exact test. All values were expressed as means ± S.E.M.

## Electronic supplementary material

Supplementary Figures
