# Supplementary material for: Running reorganizes the circuitry of one-week-old adult-born hippocampal neurons
Source: Sci Rep. 2017 Sep 7;7:10903. doi: 10.1038/s41598-017-11268-z (PMC5589841; doi:10.1038/s41598-017-11268-z)
Supplement: Supplementary file 1 — Supplementary Figures [file 41598_2017_11268_MOESM1_ESM.pdf]

Supplementary Information

Running reorganizes the circuitry of one-week-old adult-born hippocampal neurons

Nirnath Sah[1#], Benjamin D. Peterson[1#], Susan T. Lubejko[1], Carmen Vivar[2*], Henriette van Praag[1*]

[1]*Neuroplasticity and Behavior Unit, Laboratory of Neurosciences, Intramural Research Program, National Institute on Aging, National Institutes of Health, Baltimore, MD 21224, USA*
[2]*Laboratory of Neurogenesis and Neuroplasticity, Department of Physiology, Biophysics, and Neuroscience, Center for Research and Advanced Studies of the National Polytechnic Institute, Mexico City, Mexico 07360.*

[#] Equal contribution
[*] Corresponding authors

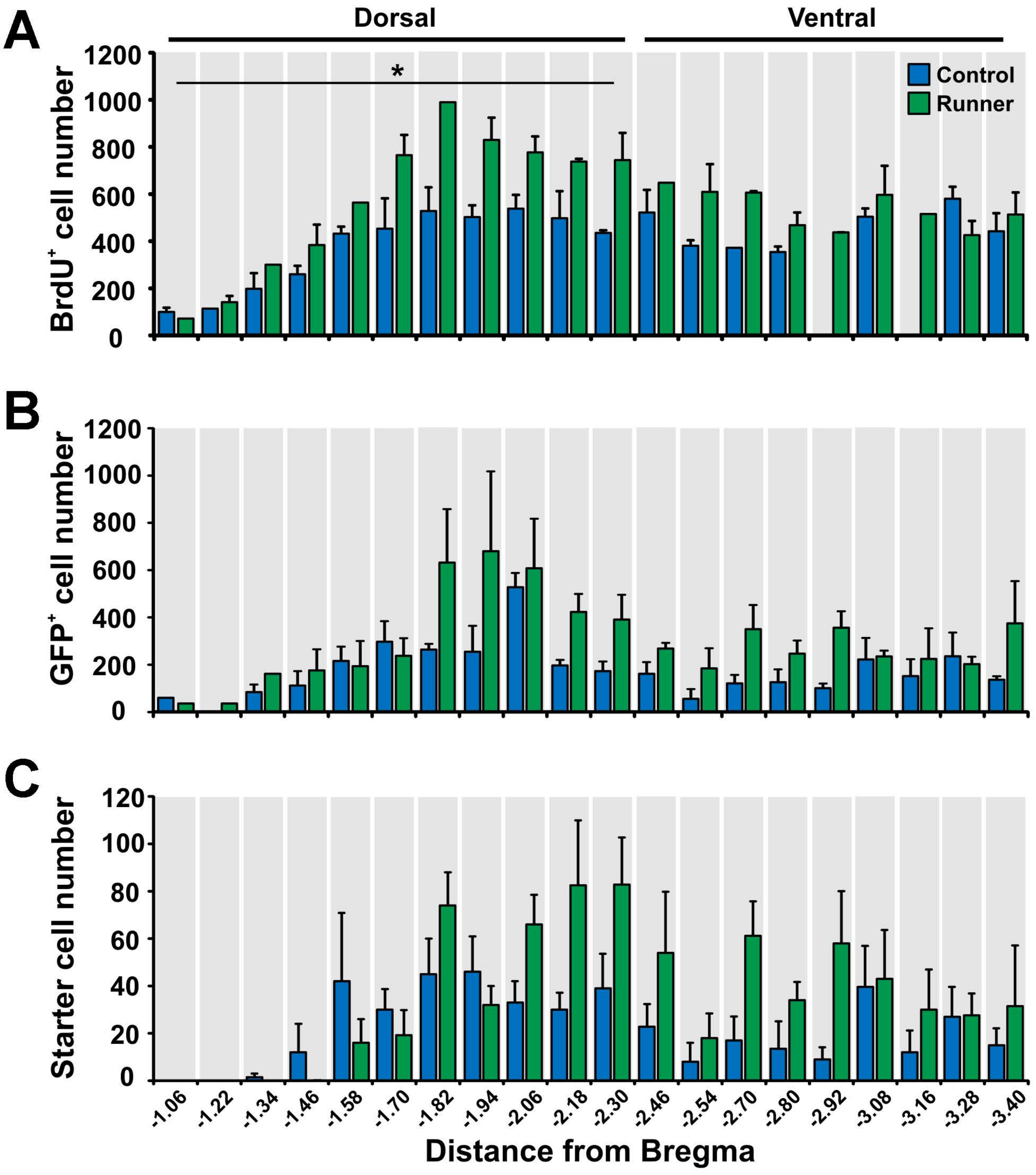
A
Dorsal
Ventral
*
Control
Runner
BrdU+ cell number
0
200
400
600
800
1000
1200
B
GFP+ cell number
0
200
400
600
800
1000
1200
C
Starter cell number
0
20
40
60
80
100
120
-1.06
-1.22
-1.34
-1.46
-1.58
-1.70
-1.82
-1.94
-2.06
-2.18
-2.30
-2.46
-2.54
-2.70
-2.80
-2.92
-3.08
-3.16
-3.28
-3.40
Distance from Bregma

Supplementary Figure 1. Running increases the number new granule cells in the dentate gyrus. (A) Dorso-ventral distribution of BrdU+ cells. Running significantly increases the number of BrdU$^+$ cells in the dorsal (1976 $\pm$ 163 cells, control; 2980 $\pm$ 218 cells, runner; $t_{(8)} = 3.43$, $P < 0.008$) but not in the ventral (2274 $\pm$ 206 cells, control; 2532 $\pm$ 204 cells, runner; $t_{(8)} = 0.77$, $P > 0.45$) dentate gyrus. (B) Retrovirally labeled GFP$^+$ cells and (C) Starter cells (GFP$^+$ retrovirus and MCh$^+$ rabies virus), throughout the rostral-caudal extent of the dentate gyrus of control and runner mice.

Supplementary Fig. 2

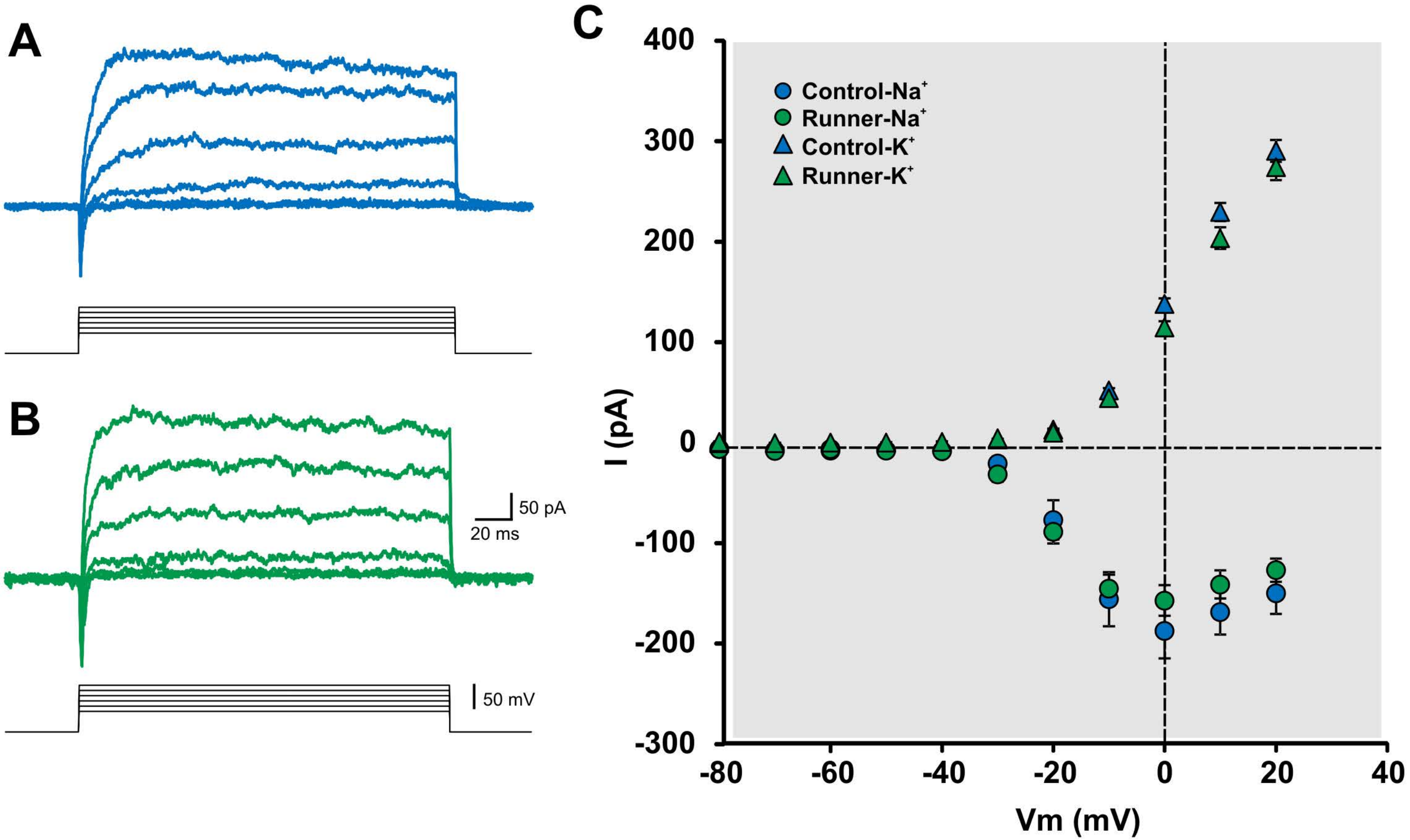

Supplementary Figure 2. Na^+ and K^+ currents of GFP^+ cells at 7-8 dpi. (A, B) Representative responses to voltage steps (-40 mV to +20 mV, 10 mV increments) of GFP^+ cells from (A) control and (B) runner mice. Traces show fast inward and slow outward currents. (C) Current-voltage (I-V) curve of peak inward current (circles) and the steady-state of the outward current (triangles) from GFP^+ cells of control and runner mice.

Supplementary Fig. 3

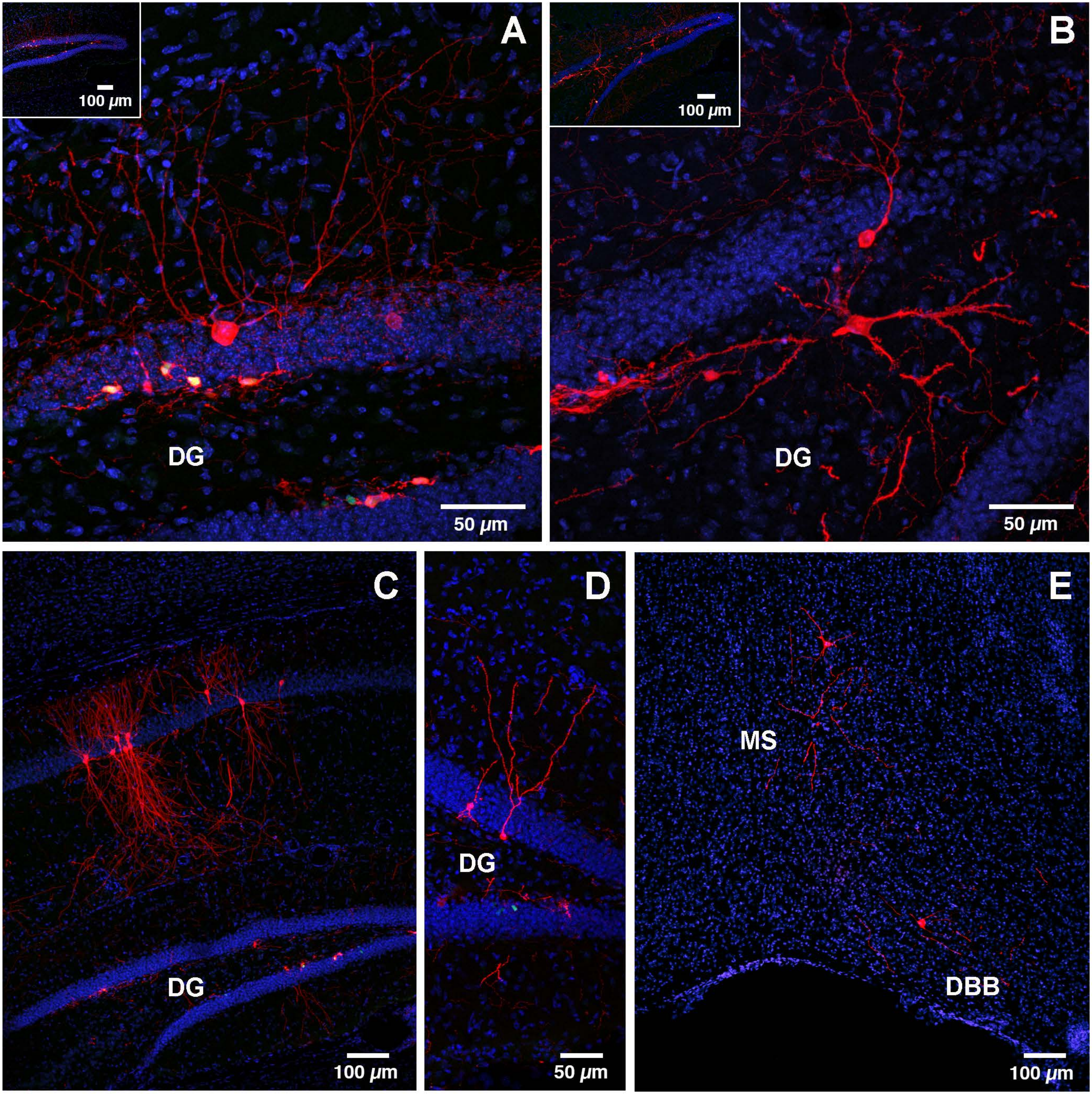

Supplementary Figure 3. Trans-synaptic tracing of immature adult-born GC inputs in 8-week-old mice. The inputs to one-week-old adult-born dentate granule cells in this cohort (n = 4, 8-week-old mice) were comparable to those observed in younger mice in this study. (A) Starter cells expressing GFP and MCh (yellow nuclei) and traced interneurons expressing MCh (red) in the dentate gyrus. In addition, traced (B) mossy cells, (C) pyramidal cells, (D) mature GCs and (E) basal forebrain cells expressing MCh were detected. DG, dentate gyrus; MS, medial septum; DBB, diagonal band of Broca. Nuclei were stained with DAPI (blue).
